# Active monitoring improves radiopharmaceutical administration quality

**DOI:** 10.3389/fnume.2023.1126029

**Published:** 2023-03-07

**Authors:** James R. Crowley, Iryna Barvi, Jackson W. Kiser

**Affiliations:** ^1^ Department of Molecular Imaging, Carilion Clinic, Roanoke, VA, United States; ^2^ Lucerno Dynamics, LLC, Cary, NC, United States

**Keywords:** extravasations, ^18^F-FDG, ^99m^Tc-MDP, quality improvement, infiltrations

## Abstract

**Introduction:**

In 2016, our center adopted technology to routinely monitor ^18^F-FDG radiopharmaceutical administrations. Within six months of following basic quality improvement methodology, our technologists reduced extravasation rates from 13.3% to 2.9% (*p* < 0.0001). These same technologists administer other radiopharmaceuticals (without monitoring technology) for general nuclear medicine procedures in a separate facility at the clinic. Our hypothesis was that they would apply ^18^F-FDG lessons-learned to ^99m^Tc-MDP administrations and that ^99m^Tc-MDP manual injection extravasation rate would be consistent with the ongoing ^18^F-FDG manual injection extravasation rate (3.4%). We tested our hypothesis by following the same quality improvement methodology and added monitoring equipment to measure extravasation rates for ^99m^Tc-MDP administrations.

**Results:**

816 ^99m^Tc-MDP administrations were monitored during 16-month period (four 4-month periods: A, B, C, D). Period A (first four months of active monitoring) extravasation rate was not statistically different from the Measure Phase extravasation rate of the previously completed PET/CT QI Project: 12.75% compared to 13.3% (*p*-0.7925). Period A extravasation rate was statistically different from Period C (months 9–12) extravasation rate and Period D (months 13–16) extravasation rate: 12.75% compared to 2.94% and to 3.43% (*p* < 0.0001). During Period C and D technologists achieved extravasation rates comparable to the longstanding manual ^18^F-FDG injection extravasation rate (3.4%).

**Conclusion:**

Our initial hypothesis, that awareness of a problem and the steps need to correct it would result in process improvement, was not accurate. While those factors are important, they are not sufficient. Our findings suggest that active monitoring and the associated display of results are critical to quality improvement efforts to reduce and sustain radiopharmaceutical extravasation rates.

## Introduction

Proper administration of a diagnostic radiopharmaceutical is essential for nuclear medicine image quality and quantification (1–8). Extravasation, an incomplete delivery of a radiopharmaceutical into the venous system, can compromise imaging, the patient's ensuing care, and sometimes lead to unnecessary additional radiation dose for repeat imaging studies. In addition, large extravasations can pose radiation harm to patients (9).

Studies suggest that an average of 15% (2 to 23%) of radiopharmaceutical administrations may result in an extravasation (5, 6, 10–14), but currently there is no routine quality control to ensure complete delivery of the radiopharmaceutical into the patient's circulation. However, with adequate training and ongoing injection quality monitoring, technologists are capable of improving injection quality significantly (15) and sustaining the results by implementing ongoing monitoring (16).

In 2016, our center participated in a multicenter ^18^F-FDG radiopharmaceutical administration QI Project, using Define, Measure, Analyze, Improve, Control methodology (15). The goal of the Project was to monitor injection quality, use analysis of factors that contribute to extravasations to guide improvements, remeasure rates in similar number of patients, and evaluate sustainability of interventions.

A quality improvement plan (QIP) was created on the basis of the analysis of contributing factors and in discussion with each technologist participating in the study. The QIP included three components: addition of an auto-injector to provide consistent infusion and flush parameters across injections, adjustment of uptake room setup to allow for improved access to left and right arms of the patient, and peer-led refresher training for venous access and injection technique. Following the QIP the technologists reduced the extravasation rates from 13.3% (Measure Phase) to 2.9% (Control Phase) (16). The 2.9% was a combination of auto injections extravasation rate (0.6%) and manual injection extravasation rate (7.1%). Ongoing monitoring ensured continued sustainability of results and further reduced manual injection extravasation rate to 3.4%.

At our clinic, general nuclear medicine procedures were performed in a different facility than PET/CT procedures, but the technologists who performed ^99m^Tc-MDP administrations were also the same ones who performed ^18^F-FDG administrations. These technologists were aware of the steps required to reduce ^18^F-FDG administration extravasations. As a result, we hypothesized that they would apply ^18^F-FDG quality improvement lessons-learned to ^99m^Tc-MDP administrations, and the resulting ^99m^Tc-MDP administration manual injection extravasation rate would not be statistically different from the ongoing manual injection extravasation rate (3.4%) in ^18^F-FDG administrations.

We tested our hypothesis by adding monitoring equipment 30 months after completing the PET/CT administration quality plan, and following quality improvement methodology, measured a baseline extravasation rate for ^99m^Tc-MDP administrations. Our findings suggest a new hypothesis: active monitoring of radiopharmaceutical administrations is critical to ensuring radiopharmaceutical administration quality.

## Materials and methods

The Institutional Review Board of Carilion has determined that quality improvement efforts for ^18^F-FDG do not meet the definition of research as defined by the federal government in 45 CFR 46.102(d) and therefore, no patient consent was required. No protected health information was collected or transmitted. Every patient undergoing routine ^99m^Tc-MDP bone scans was eligible to be included in the QI methodology. Patients followed standard clinical preparations for SPECT/CT imaging and were not exposed to additional radiation during this Project.

**Figure 1:**
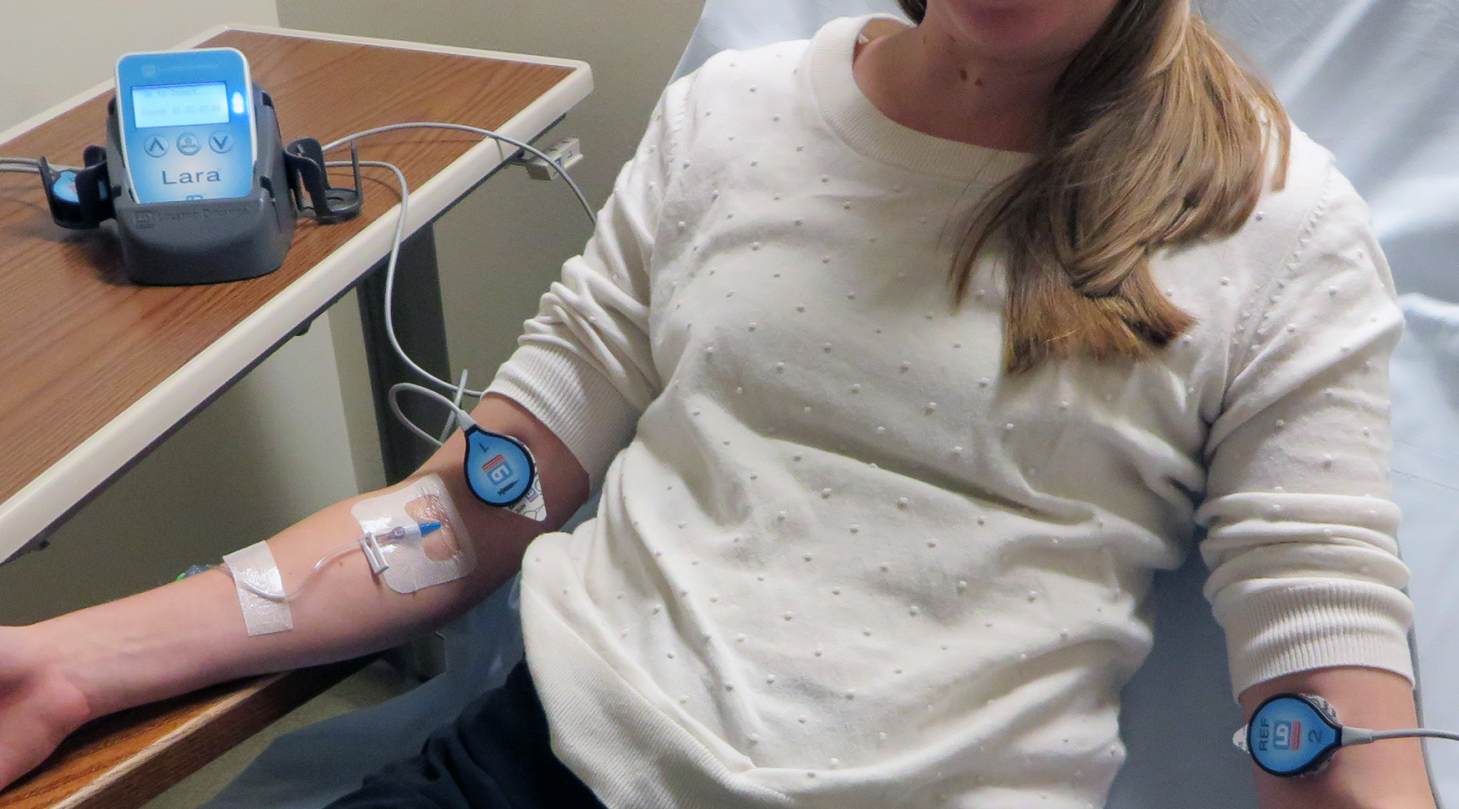
The Lara System consists of 2 scintillating sensors, 2 adhesive pads, reader, docking station and software. Sensors are placed on injection and reference arm of the patient. Real-time counts are visible on reader's display.

For 30 months after completion of the ^18^F-FDG quality improvement project we did not actively monitor the quality of ^99m^Tc-MDP administrations. We then introduced a Lara® System (Lucerno Dynamics, Cary, NC) designed for capturing ^99m^TC emissions to help clinicians determine the quality of MDP administrations. The Lara® System is comprised of gamma scintillating sensors, adhesive pads, a reader, and software (Figure 1). After technologists gain venous access, they place the sensors on the injection arm and in a mirrored location on the other arm. The sensors identify the presence of excess radiopharmaceutical near the injection site and are connected to a reader that provides real-time display of activity in each arm. The reader also stores data and later transmits the recorded scan to a server for processing of time-activity curves (TACs).

Bone SPECT images were acquired with a Siemens Intevo Bold (Siemens Medical Solutions USA, Inc.) after standard injected activity of 25mCi ^99m^Tc-MDP using a LEHR collimator and matrix size of 256 × 256 with a zoom of 1. SPECT data were acquired using 30 views in a noncircular (body contour) orbit with an acquisition time of approximately 20 s per view. For CT Acquisition, data were acquired using 130 kVp with CareDose 4D and pitch of 1.5 and detector settings of 16 × 1.2 mm with 3 mm slice thickness.

### Statistical analysis

Statistical analysis was performed using the JMP 16.0 statistical software package (SAS). Unadjusted extravasation rates were calculated by dividing the number of extravasations by the number of injections. The Fisher's Exact test and Two Sample Test were used to compare increase or decrease in extravasation rates in groups before and during active monitoring. All statistical tests were 2-sided, and a threshold *P* value of less than 0.05 was considered statistically significant for rejection of the null hypothesis. Chi-square test was used to identify contributing factors in each period as well as to identify whether the difference in extravasation rates among those factors is statistically significant.

## Results

**Figure 2:**
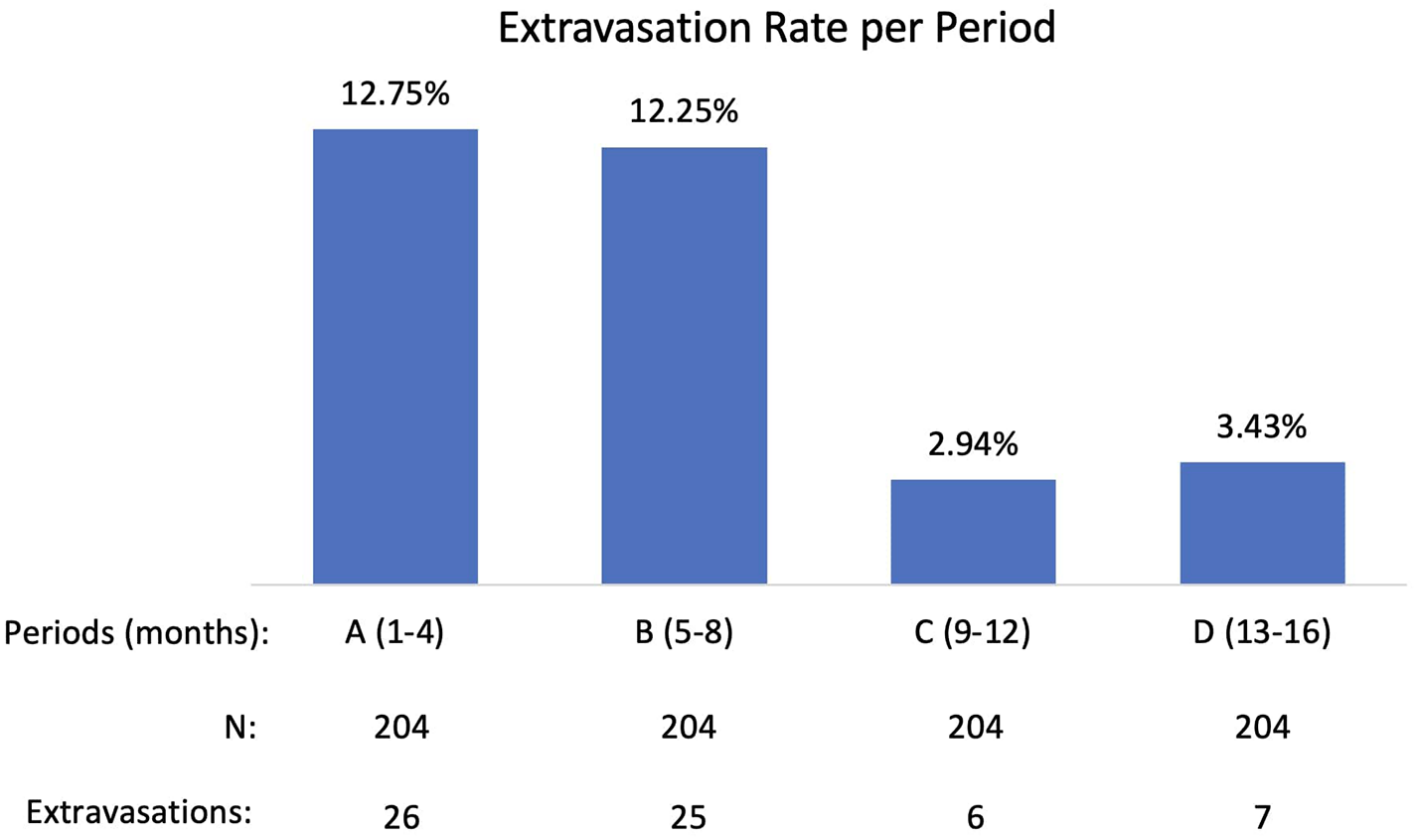
Extravasation rate reduction during four periods.

Data were collected on 816 administrations during 16-month period and the extravasation rate was found to be 7.84%. A closer examination of the 16-month (four 4-month periods: A, B, C, D) and potential contributing factors revealed that a significant reduction in extravasation rate had occurred over these periods (Figure 2).

Period A (first four months of active monitoring) extravasation rate was not statistically different from the Measure Phase extravasation rate of the previously completed PET/CT QI Project: 12.75% compared to 13.3% (*p*-0.7925). Periods C (months 9–12) and D (months 13–16) extravasation rates were statistically lower than period A extravasation rate: 2.94% and 3.43% (*p* < 0.0001) compared with 12.75%. After analyzing contributing factors and implementing improvements, technologists achieved extravasation rates during Period C and D comparable to the longstanding manual ^18^F-FDG injection extravasation rate (3.4%).

Our contributing factors analysis suggests that during Period A, injection site location, venous access technique, and needle size, were associated with higher extravasation rates (Table 1). Further analyses suggest that when technologists stopped using the venous access technique of straight sticks (Table 2) and 23-gauge needles (Table 3), those factors were no longer associated with extravasation rates in Period D.

**Table 1:** Period A contributing factors.

Effect	*p* value
Wrist injection location is associated with higher predicted probability of extravasation when compared to an antecubital	0.0312
Straight stick technique is associated with higher predicted probability of extravasation when compared to IV technique	0.0001
23-gauge needle is associated with higher predicted probability of extravasation when compared to 22-gauge needle	0.0001

**Table 2:** Straight stick extravasation rate compared to IV access technique extravasation rate.

Period	Straight Stick			IV Access		
	Number of injections	Number of extravasations	Extravasation rate	Number of injections	Number of extravasations	Extravasation rate
A	44	11	25.00%	160	15	9.38%
B	26	9	34.62%	178	16	8.99%
C	21	2	9.52%	183	4	2.19%
D	0	0	0.00%	204	7	3.43%

**Table 3:** 23-Gauge needle extravasation rate compared to other needle gauge extravasation rates.

Period	23 Needle Gauge			Other Needle Gauge (20-, 22-, 24-, 25-)		
	Number of injections	Number of extravasations	Extravasation rate	Number of injections	Number of extravasations	Extravasation rate
A	19	4	21**.**05%	185	22	11**.**89%
B	15	5	33**.**33%	189	20	10**.**58%
C	3	0	0**.**00%	201	6	2**.**99%
D	0	0	0**.**00%	204	7	3**.**43%

## Discussion

Our hypothesis that problem awareness and solution availability from the previously completed quality improvement project would result in radiopharmaceutical administration process improvement was not accurate. While those factors are important, they are not sufficient. At our center, active monitoring and the associated display of results have proven to be critical to quality improvement efforts to reduce and sustain radiopharmaceutical extravasation rates. Active monitoring provides information necessary to determine contributing factors and when combined with active management review of displayed results, leads to improved administration quality. Our findings related to active monitoring are also supported by another center's quality improvement results^1^.

While ongoing active monitoring may be necessary for sustained quality administrations, it has many other implications.

### Regulatory implications

In 2020 members of nuclear medicine community had an opportunity to share their position on diagnostic extravasations with the Nuclear Regulatory Commission (NRC). The NRC had requested public comments on a petition to eliminate an internal NRC policy that exempts extravasations from medical event reporting, even if the extravasation's absorbed tissue dose exceeded the reporting limit. As part of the request for public comments, the NRC asked: “Do you expect that monitoring for extravasation and reviewing the results would improve radiopharmaceutical administration techniques at medical use licensee facilities?” The leaders of the Society of Nuclear Medicine and Molecular Imaging (SNMMI), the organization that provides guidelines and standards for molecular imaging and nuclear medicine practice, stated that regular in-service education of those individuals who administer radiopharmaceuticals was important, but that “monitoring is not expected to improve administration techniques”^2^. Our findings and the well-documented “observer effect” experienced by our technologists suggest the opposite: monitoring will improve administration techniques.

### Quality improvement implications

Measurement and identification of contributing factors are critically important for process improvement. Nuclear medicine extravasation rates have been difficult to measure without active monitoring. These measurement difficulties are related to a few factors. When extravasated, the small injection volumes of non-vesicant radiopharmaceuticals do not cause immediate, visible changes to the overlying skin near the injection site, nor immediate pain to patients. Additionally, injection sites are routinely outside of the standard imaging field of view for non-MDP injections (10). When diagnostic extravasations go undetected, and centers do not understand contributing factors, administration quality is difficult to improve. Active monitoring enables measurement and provides public feedback specific to technologist/technologist teams based on analyses of contributing factors, leading to process improvement efforts.

### Dosimetric implications

Routine diagnostic procedures, including bone scans, are often viewed as low risk to patients. However, recent findings show that large extravasations of diagnostic radiopharmaceuticals can result in high doses to the tissue leading to adverse tissue reactions (9, 17). In such cases timely measurements of radioactivity can protect both patients and institutions, assisting in determining the need for repeat imaging and patient follow-up. In order to assess absorbed dose to tissue, the following factors are important: initially extravasated activity, emission types and energies, the mass of affected tissue, and the rate of biological clearance (9). Active monitoring promptly identifies extravasations that require dosimetry. It also helps to determine biological clearance and initially extravasated activity (9). Active monitoring can reveal patient- or procedure-specific insights. For example, due to the chemical properties of ^99m^Tc-MDP, biological clearance can be limited. Additionally, extravasations of ^99m^Tc-MDP, which is commonly injected using a straight stick with no saline flush, can result in a small volume of highly concentrated radiopharmaceutical.

### Precision medicine implications

Precision medicine is becoming more important in radiology and nuclear medicine. Variability in clinical interpretation of images limits progress toward precision medicine. Extracting reproducible, quantitative standardized uptake value (SUV) results from scans is important in reducing variability and becoming an expected performance measure (18). Obtaining accurate SUV measures requires attention to all potential sources of variance. Extravasation can be a major source of variance. Active monitoring of injection quality can reduce this variance.

### Theranostic implications

The use of radiotherapy is growing worldwide. These therapies typically require administrations of high activity alpha- and beta-emitting energies. Often times these therapies use isotopes with longer half-lives which increase their effectiveness. Many radiotherapeutic procedures do not include imaging until the following day or days. As a result, an extravasation of administered activity can go undetected and result in a high absorbed dose to tissue (19, 20). Active monitoring of the radiotherapeutic administration raises technologist awareness and allows immediate application of mitigation strategies to minimize absorbed dose to tissue.

## Conclusions

Our initial hypothesis that awareness of a problem and the steps needed to correct would result in process improvement was incorrect. In our center, technologists trained in QI methodology had not applied lessons-learned from their ^18^F-FDG PET/CT QI Project to ^99m^Tc-MDP administrations. However, by requiring active monitoring and displaying results, the trained technologists determined contributing factors and reduced extravasation rates significantly. These findings are consistent with our previous experience in PET/CT QI findings from another center, and the well-known observer effect phenomenon. These findings also suggest that centers that want to improve their radiopharmaceutical extravasation rates can benefit by implementing active monitoring.

## Data Availability

The original contributions presented in the study are included in the article/Supplementary Material, further inquiries can be directed to the corresponding author.

## Footnotes

^1^Allison Fulp, Shane Masters, Anita Thomas, Jean-Luc Urbain, Jonathan Richardson, Paige Bennett. *Improving the quality of PET radiopharmaceutical injections: Our lessons learned.* Wake Forest School of Medicine at SNMMI Annual Meeting. Available at: https://lucerno.com/wp-content/uploads/2023/02/WFU_Poster.pdf

^2^Available online at: https://www.regulations.gov/comment/NRC-2020-0141-0428.
